# Objectively identifying landmark use and predicting flight trajectories of the homing pigeon using Gaussian processes

**DOI:** 10.1098/rsif.2010.0301

**Published:** 2010-07-23

**Authors:** Richard Mann, Robin Freeman, Michael Osborne, Roman Garnett, Chris Armstrong, Jessica Meade, Dora Biro, Tim Guilford, Stephen Roberts

**Affiliations:** 1 Department of Engineering Science, University of Oxford, Oxford, UK; 2 Department of Zoology, University of Oxford, Oxford, UK; 3 Computational Ecology and Environmental Science, Microsoft Research, Cambridge, UK; 4 Department of Animal and Plant Sciences, University of Sheffield, Sheffield, UK

**Keywords:** animal movement, avian navigation, pigeon, Gaussian process, landmarks, flight

## Abstract

Pigeons home along idiosyncratic habitual routes from familiar locations. It has been suggested that memorized visual landmarks underpin this route learning. However, the inability to experimentally alter the landscape on large scales has hindered the discovery of the particular features to which birds attend. Here, we present a method for objectively classifying the most informative regions of animal paths. We apply this method to flight trajectories from homing pigeons to identify probable locations of salient visual landmarks. We construct and apply a Gaussian process model of flight trajectory generation for pigeons trained to home from specific release sites. The model shows increasing predictive power as the birds become familiar with the sites, mirroring the animal's learning process. We subsequently find that the most informative elements of the flight trajectories coincide with landscape features that have previously been suggested as important components of the homing task.

## 1. Introduction

The domestic homing pigeon (*Columba livia*) is the canonical test species for scientific studies of avian navigation. The past decade has seen an experimental paradigm shift in the study of pigeon homing since the development of micro-GPS logging devices small enough to be carried by a bird in flight [1–3]. The use of these devices has enabled researchers to obtain data of very high spatial and temporal resolution about the bird's position during flight and has revealed hitherto unsuspected phenomena. These include the propensity to follow roads and other strong linear features in the landscape [3–6] and the tendency to form idiosyncratic habitual routes back to the loft when released repeatedly from a single site [4,7].

Experiments suggest that pigeons have a very robust loyalty to their habitual routes once formed. Pigeons displaced up to 1.5 km perpendicular from their habitual route before release are observed to recapitulate the established habitual route, rapidly rejoining the original path [4]. This implies a non-compass-based orientation since a compass bearing from the new location would direct them towards the home loft rather than directly back to the habitual route. The habitual route has also been shown to be robust under manipulation of known compass mechanisms. Birds whose azimuthal sun-compass mechanism [8] has been disturbed by clock-shifting techniques perceive an effective compass bearing that is rotated relative to reality. Birds navigating by a clock-shifted compass typically fly at a displaced bearing to their non-clock-shifted flights ([9], ch. 5). However, experiments with birds that have previously formed habitual routes from familiar release sites show that clock-shifted birds are able to faithfully follow the habitual route they have previously formed with only minor displacements [10]. Pigeons can also form habitual routes when fitted with a magnetic material to disrupt their magneto-sensory compass mechanism [7].

These discoveries are persuasive evidence that orientation in the familiar area is controlled principally by visual recognition. Pilotage, defined as flying between a series of fixed points in sequence, has been posited as the most likely homing mechanism [4], probably implying a memorized route between the release site and the home loft encoded by the locations of fixed geographical landmarks. Although several studies have observed pigeons following particular features [4,5], further understanding of how information from the landscape controls flight behaviour has remained out of reach because experimental manipulation of landmarks on such a scale is not practical. Determining the locations of important landmarks in an objective manner is a crucial step towards discovering the visual features that birds use to orient themselves in familiar environments.

The flight paths of homing pigeons can now be routinely measured using GPS devices, which record the position of the bird once a second for the entire duration of the flight. Here, we consider these flight paths as random variables, which we aim to model through an appropriate probability distribution, using Gaussian processes (GPs) as a framework for performing inference over function-valued variables. In this study, we show how a GP model can quantitatively predict the future flight paths of a trained individual bird based on observations of its previous flights. By isolating the points during the past flights that allow for the best predictions of future flights, we demonstrate an objective algorithm for automatically detecting navigational landmarks.

### 1.1. Gaussian processes

GPs are a powerful and flexible framework for performing inference over functions [11]. The distribution over the function, *f*(·), is specified by a mean function, *m*(·), and a covariance function, *k*(·,·), that determines the correlation between disparate locations on the function. If *f*(*t*) is a draw from a GP, then any finite number of function values, *f*(***t***), evaluated at a set of inputs, ***t***, has a multi-variate Gaussian distribution (represented as standard by 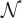)1.1
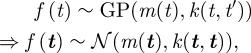
where *k*(***t***, ***t***) indicates the matrix evaluation of *k*(*t*, *t*′) for all possible pairs of components of the vector of input values, ***t***. The prior mean function is chosen to accurately represent the prior knowledge of the function (typically being specified by a symmetry in the model). The covariance kernel is chosen to represent prior beliefs about the dynamical structure of the function—how the function values change with varying inputs. In this paper, we will consider only the following stationary Matérn covariance [12],1.2
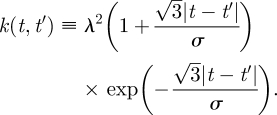


The kernel, *k*(*t*, *t*′), represents the strength of correlation between function values for inputs separated by |*t* − *t*′|. It is a decreasing function of |*t* − *t*′|; therefore, closely spaced values of the function are highly correlated and widely spaced values are roughly independent. The adjustable parameters, *λ* and *σ*, are, respectively, termed the output scale and the input scale. The output scale specifies the absolute degree of variation in the function values—larger output scales mean that the function values can vary more widely from the prior mean. The input scale specifies a characteristic correlation length that determines how smoothly the function varies. Larger values of the input scale correspond to smooth, slowly varying functions. In the case of a moving object, such as a pigeon, larger input scales correspond to lower average accelerations.

The key property of this covariance function is the monotonic decay with increasing values of |*t* − *t*′|, which implies an adjustable degree of smoothness in the functions sampled from this GP. Our results are not sensitive to the exact form of the function, but this specific form is selected from within the general Matérn class of functions to maximize the marginal probability of pigeon flight trajectory data. For a discussion of alternative covariance functions, see [11].

This specifies a *prior* distribution over the function before we make any observations. Now assume that we have observations, *f*(***t***_***D***_), and are interested in making predictions about the value of the function, *f*(t_*_), at inputs t_*_. We require the *posterior* distribution over *f*(*t*), the probability distribution over the function conditioned on the data we have already seen, which is given by1.3
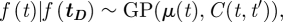
with updated mean and covariance matrices1.4
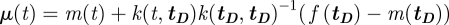
and1.5
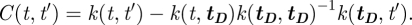


Thus, our beliefs about the value of *f*(*t*) are altered in proportion to how far the observed function values are from our prior expectations and the strength of correlation between the value of the function at differing input arguments.

## 2. Model

A pigeon's loyalty to its habitual route makes it *predictable*. We suggest that observed flight trajectories represent imperfect attempts to replicate an *unseen and never seen* idealized habitual route. Variation around the idealized habitual route is uncorrelated between different flight trajectories. Therefore, we aim to learn about the structure of the underlying habitual route and the scale of variation around it from these imperfect observations. Here, we give the mathematical construction of that model, which culminates in the posterior distribution for future flight trajectories conditioned on previously observed flights by the same bird. Supplementary to this paper, we also provide an implementation of this model as a Matlab toolbox. The toolbox can be downloaded from the Oxford Navigation Group (OxNav) website at: http://oxnav.zoo.ox.ac.uk/downloads.

Each flight trajectory, *x*_*i*_(*t*), is a two-dimensional continuous function of time. In our model, an *observed* flight trajectory, *x*_*i*_(***t***_***i***_), is a finite vector of position observations from that function at input times, ***t***_***i***_. We model this as a sample from a GP, with a mean, *h*(***t***_***i***_), which represents the habitual route, and a covariance matrix, *k*_*ϕ*_(***t***_***i***_, ***t***_***i***_), which determines both the scale of variation around the habitual route and the smoothness of the trajectory (parametrized by input and output scales, *ϕ* ≡ {*λ*,*σ*}). Multiple trajectories will be assumed to have been generated from a common idealized habitual path—mathematically, this means they are identically and independently distributed from this GP, sharing a common mean function, *h*(*t*), representing the idealized habitual route. The finite precision of the GPS device introduces observation error, which we model as isotropic Gaussian noise with variance *η*^2^. The resolution of a typical GPS device is within 5 m, which informs the prior distribution over this hyper-parameter. The observation noise is incorporated by the addition of the identity matrix, represented by the Kronecker delta function *δ*(***t***_***i***_, ***t***_***i***_), to the covariance. With these considerations, the distribution of a single flight path, *x*_*i*_(***t***_***i***_), conditioned on knowing the habitual route, *h*(*t*), is given by2.1
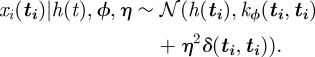


Here, the subscript *i* indexes the flight number—therefore, ***t***_***i***_ represents the vector of observation times for flight *i*. The input variable *t* is constrained to lie between zero and 1, with zero representing the release and 1 representing collection of the bird at the loft. Thus, the time index of the flights is a proportion of the total flight duration.

A GP prior distribution is placed over the common habitual route, *h*(*t*). We argue that, having disregarded any knowledge of the environment, symmetry requires that this distribution be centred on the straight ‘beeline’ route, *s*(*t*), between the release site and the loft. The habitual route has its own dynamical structure parametrized by the covariance kernel, *k*_*θ*_(*t*, *t*′), where *t* and *t*′ are any time indices for the habitual route function. Similarly to the covariance for the observed flight path, the covariance kernel for the habitual route has its own hyper-parameters (input and output scales), *θ* = {*λ*_*h*_,*σ*_*h*_}. The habitual route is an unobserved process and thus includes no observation noise, but we still have uncertainty in the value of *h*(*t*) since we cannot observe it directly. At this stage, the habitual path is a continuous function. The distribution over *h*(*t*) is therefore a GP rather than a multi-variate Gaussian distribution and is given by2.2
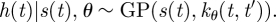


Since the habitual path is never observed, we integrate over all possible values to obtain a distribution over sets of trajectories sharing a common, unknown *h*(*t*),2.3
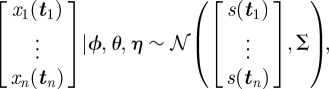
with a combined covariance *Σ*:2.4
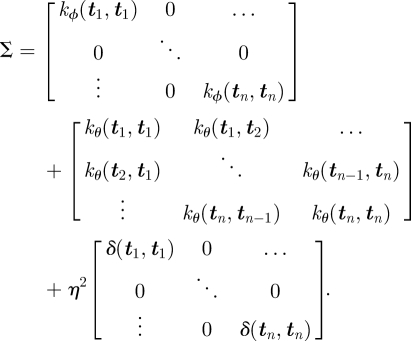


The three terms in equation (2.4) correspond to three distinct facets of the model. The first is the covariance owing to the variation of each trajectory around the habitual route. This is a diagonal block matrix since this variation is uncorrelated across different trajectories. The second term corresponds to the covariance associated with the shared habitual route. Finally, the third term is due to the observation noise associated with measuring the pigeon's position with the GPS device.

The distribution over an as-of-yet unseen flight path, x_*_(***t***), for any set of observation times, ***t***, can now be obtained by application of equation (1.3),2.5
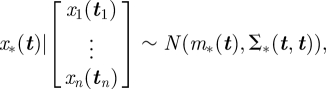
with updated mean and covariance functions given by2.6
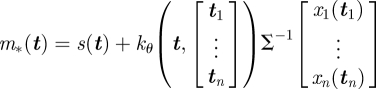

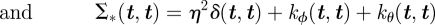
2.7
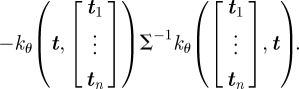


In the development of this model, we have used the proportion of the total flight duration as a latent variable that maps to the observable output of the bird's position. It should be noted that this imposes some restrictions on the power of the analysis. For example, if a bird leaves its habitual route to forage for food or to investigate some other salient factor such as the presence of another bird, before subsequently returning to the route, the posterior distribution may record this as a low probability flight because the bird is not where it is expected to be at a given point in time. This can be overcome to some degree by pre-processing data to remove sections where the bird is motionless, but, within the current framework, this effect is not entirely removable. As we will discuss, this will impose a conservative restriction of the number of waypoints identified from a set of trajectories.

### 2.1. Implementation

We use Bayesian marginalization, through Metropolis–Hastings Markov-chain Monte Carlo (see, for example, [13], ch. 29), for an honest propagation of the uncertainty associated with the hyper-parameters *ϕ*, *θ* and *η*. One thousand samples were generated from the posterior distribution of the GP hyper-parameters. The generated samples were then used to numerically integrate over the hyper-parameters in calculating the conditional probability of the test data (future flights) from equation (2.5).

## 3. Results

### 3.1. Predicting flight trajectories

**Figure 1.**
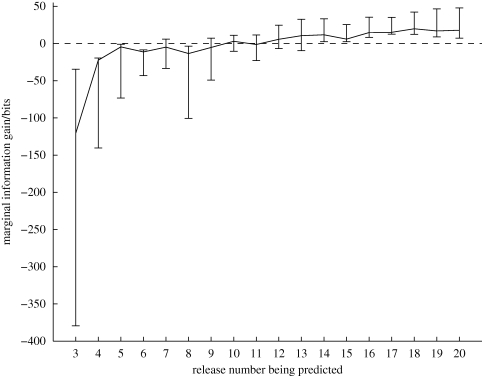
Marginal information gain (MIG) in predicting each flight trajectory from its two immediate predecessors. The figure shows the median value averaged over 31 birds, along with the interquartile range (IQ) range. Higher MIG represents greater predictability. MIG values above zero indicate that the flight trajectories are more likely as a set than as independent observations. Solid line, median with IQ range.

We collated previously collected data [7,14] from 31 birds during training flights from four distinct sites around the Oxford Field Station (§4.1). Each bird was released 20 times from its selected release site and its flight home recorded using a GPS logger. Using equation (2.5), we took consecutive pairs of flight trajectories and used them to predict the trajectory of the next flight (e.g. predicting the trajectory of the third release based on the trajectories of the first two flights). We compared this with the prior probability of the subsequent trajectory to give a metric of predictability using marginal information gain (MIG), defined as3.1
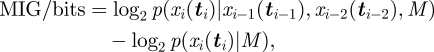
where *M* represents our model described above, *x*_*i*_ (***t***_*i*_) is the flight path being predicted and *x*_*i*−1_(***t***_*i*−1_) and *x*_*i*−2_ (***t***_*i*−2_) are the two most recent flight paths before the predicted flight path. Values of MIG above zero indicate predictable behaviour; the flight trajectory is more likely in the light of observations than it was *a priori*. Conversely, negative values of MIG indicate the non-existence of a habitual route; the flight paths are more probable as independent rather than as correlated variables since correlation through the habitual route implies low inter-flight variation. As a result, the habitual route model over-fits and makes poor predictions where flight paths show large-scale inter-flight variation. Figure 1 shows the MIG averaged (median) over the 31 birds as a function of the flight number being predicted, along with error bars indicating the interquartile (IQ) range to represent the scale of variation around the median.

The clear increase in predictability in figure 1 is a confirmation of route-learning behaviour from an information theoretic perspective. The very low predictability of the first few flights is a consequence of the extremely variable nature of the first flight of many birds. This is an expected result of the initial unfamiliarity of the birds with the release site. Earlier studies have shown that birds released at unfamiliar sites tend to circle the release site after being released and subsequently home along highly disordered trajectories (see figures in [7]). After only one previous experience of the release site this effect is often greatly reduced, explaining the substantial increase in predictability in the first few flights. After this early naivety is overcome, the trend becomes a steady but gradual increase in predictability continuing until the final flight, indicating a continued increase in the birds' fidelity to their habitual routes.

### 3.2. Identifying landmarks

We use a forward-selection (‘greedy’) algorithm to determine an optimal subset of previous observations, using the observations at the same points in time from each previous flight. Forward selection aims to pick a subset of observations that maximizes the marginal likelihood of each of the flight trajectories, using the same formalism as the MIG criterion in equation (3.1). Let *x*_*i*_(***t***^*m*^) refer to *m* observations of flight *i* at times ***t***^*m*^. At each iteration, we add another observation to the subset to maximize the MIG,3.2
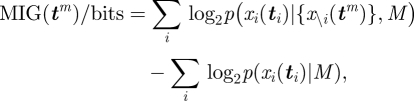
where {*x*\_*i*_(***t***^*m*^)} indicates all paths except path *i* (we sum the calculation over all paths to reduce the impact of outliers). In this calculation, the distribution of the hyper-parameters, *ϕ*, *θ* and *η*, is not inferred from the subset of data, since this would force the algorithm to choose waypoint locations so as to minimize the uncertainty on the hyper-parameters, rather than capturing the spatial information. For example, the selection algorithm may select a series of closely spaced data points in order to minimize uncertainty over the input-scale hyper-parameters, which would provide little spatial information. We aim to mimic as closely as possible the way the pigeons use a set of geographical locations to reproduce their habitual route. A pigeon does not need to use these positions to ‘learn’ model hyper-parameters, since these represent flight characteristics intrinsic to the bird. Therefore, we marginalize over the hyper-parameters using the prior distribution. We note that we obtain similar results by marginalizing over the hyper-parameters using the posterior distribution inferred from the complete dataset, suggesting that the algorithm is not overly sensitive to the hyper-parameter distribution.

The number of waypoints can be estimated by Bayesian model selection [13,15–17]. At each iteration, when considering the next waypoint, we perform model selection between a model using only the waypoints already selected (*M*_0_), and a model that uses both these waypoints and the additional ‘free’ waypoint we are considering adding (*M*_1_). We marginalize over the position of this new waypoint while keeping those already selected fixed. This mirrors the ‘greedy’ selection process, which considers waypoints to be fixed once they have been selected. If the Bayes factor (BF) for these two models is in favour of the model containing the additional waypoint, we place the waypoint at the optimal location and proceed to the next iteration. Otherwise, we stop and retain the waypoints selected so far. The BF for making this decision is given in terms of the MIG by the following equation:3.3
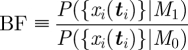
3.4
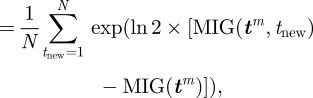
where *N* is the number of possible waypoint locations (we restrict this to 100 in our implementation) and {*x*_*i*_(***t***_*i*_)} is the complete dataset. If BF is less than 1, the evidence favours *M*_0_ and we do not add the next waypoint—the algorithm stops. The marginalization over all possible positions for the new waypoint is important to avoid over-fitting. If only the most probable position is considered, the analysis fails to honestly incorporate the uncertainty associated with that position and subsequently chooses too many waypoints since there is no cost associated with the addition of these extra parameters. This is the Bayesian interpretation of the famous principle of Occam's Razor (e.g. [16], ch. 24).

**Figure 2.**
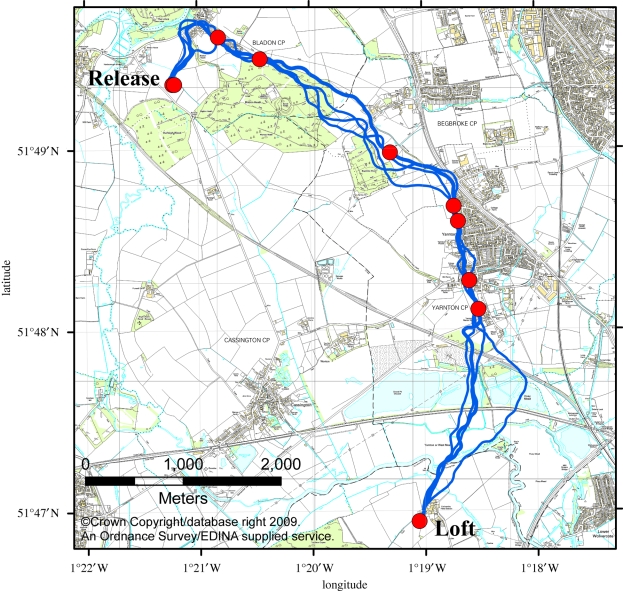
Case study of the landmark identification algorithm for a single bird released at the Bladon Heath site. The five flight trajectories used for classification are shown (blue lines), along with the 10 identified landmarks (red circles). Identified landmarks occur preferentially at the boundaries of forests and villages, in addition to the release point and the loft.

**Figure 3.**
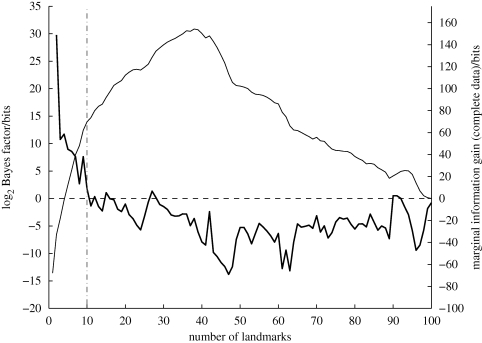
Determining the optimum number of landmarks using the BF. The plot shows the log BF (heavy line) and the MIG (light line) as more landmarks are added to the Bladon Heath case study in figure 2. The number of landmarks are selected once the log BF falls below zero, as indicated by the vertical dashed line. In this case, 10 landmarks are selected.

Figures 2 and 3 show an example of running this algorithm over the data from a single bird. Figure 2 shows the identified waypoints, along with the five flight trajectories used to identify them, plotted on an ordnance survey map of the underlying landscape. Figure 3 shows the logarithm of the BF and the MIG metric, as functions of the number of waypoints. The number of waypoints is determined by the first point at which the logarithmic BF is below zero, as this indicates that the addition of the next waypoint reduces the probability of the data.

Inspection of the identified waypoints in this case points to a number of striking visual features in the vicinity. The first identified waypoint is at the village of Yarnton, which is positioned at the apex of the flight trajectories and therefore does most to define the shape of the habitual route. Further waypoints are positioned over Bladon village, near the release site, and along the boundaries of the forests between Bladon and Yarnton. This corresponds to known behavioural facets of pigeon orientation. Kiepenheuer [18] and Wallraff [19] showed that pigeons released from unfamiliar sites showed a directional bias towards villages and forests in the vicinity, and pigeons may avoid *crossing* forested areas, for reasons that are not yet fully understood but may be related to the potential saliency of the landscape as a visual memory. Therefore, a viable hypothesis for how this route and the associated waypoints were selected is that the pigeon was initially attracted towards the village at Bladon; once there, it was prevented from directly flying towards the home loft by the obstacle of the forests and therefore flew along the boundary of these; this brought it into visual range of Yarnton village, which it was attracted to before flying home. These initial biases then determined the regions of the landscape from which it could select navigational landmarks.

**Figure 4.**
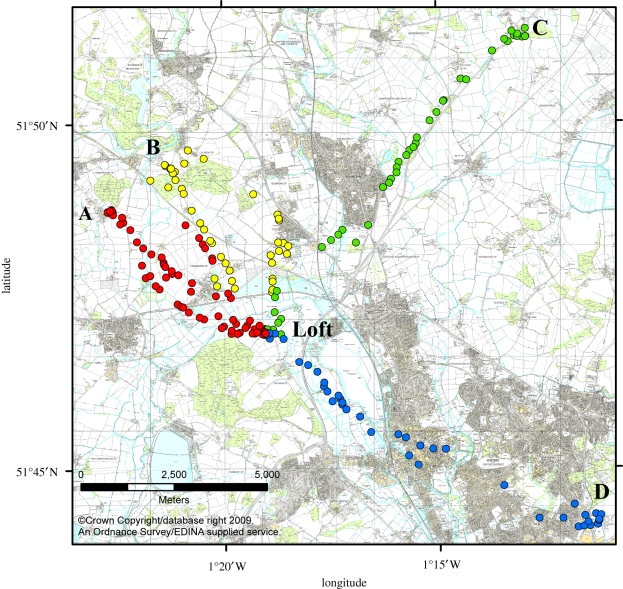
Landmarks identified from the four experimental release sites. Landmarks are colour-coded according to the release site from which they were identified. The four release sites are labelled as follows (with number of contributing birds): ‘A’, Church Hanborough (eight birds); ‘B’, Bladon Heath (seven birds); ‘C’, Weston Wood (eight birds); ‘D’, Horspath (eight birds). Landmarks are identified in similar locations from birds released from different sites, particularly in the region between Church Hanborough and Bladon Heath, indicating that the underlying features are sufficiently arresting to attract birds approaching from differing directions. Landmark use is low in urban regions, such as within the city of Oxford (bottom right) and the large village of Kidlington (top centre).

Figure 4 shows all the identified waypoints from the 31 birds used in this study, colour-coded according to the release site. The four release sites and the home loft are indicated. A notable feature of this image is the relatively low density of waypoints over urban areas. As noted in the case study, pigeons seem attracted towards small urban areas, such as villages. However, they seem either unwilling to cross them or—if avoiding them entirely is unfeasible—they form very few waypoints within them. The release site at Horspath was originally selected to explore the behaviour of pigeons over urban landscapes by forcing them to fly over the suburbs and centre of Oxford [14]. As can be seen here, the density of waypoints is very low in suburban Oxford, near the release site, increases slightly in the centre of the city and increases more dramatically once the trajectories leave the city and enter the rural area between Oxford and the home loft.

The identified waypoints from the Weston Wood release site reproduce earlier findings that pigeons use the major road leading from the release site in the direction of the home loft. The highest density of waypoints occurs when the road changes direction—at this point, most of the pigeons' habitual routes leave the road and become more variable. Again there is an absence of waypoints within the urban area that intersects the natural flight corridor.

The waypoints close to the release site at Bladon Heath are a notable example of the result of pigeons' reluctance to cross forested areas. These pigeons form waypoints all around the edge of the two forested areas close to the release site, and through a narrow unforested partition, but rarely fly directly across the forested areas.

The waypoints identified from releases at Bladon Heath and Church Hanborough show a substantial element of overlap, as do waypoints identified from the other sites within the region around the home loft where the routes converge. This is persuasive evidence that some underlying feature of the landscape is sufficiently visually arresting to attract not only different pigeons, but also pigeons released from different sites.

**Figure 5.**
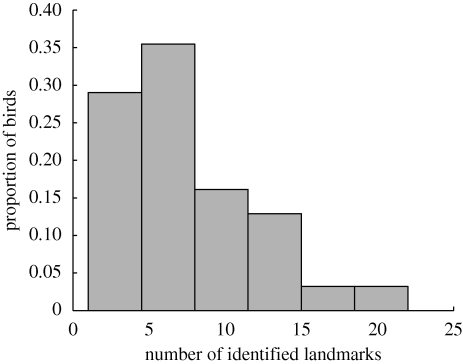
Histogram of the number of landmarks identified for each bird. The median value is seven, with a maximum of 22 and a minimum of one. A large majority (77%) of birds use 10 landmarks or fewer.

A key question regarding the use of waypoints is the number of such points a bird will typically require to memorize its route. In this study, we find a median value of seven for the number of waypoints used, across all 31 birds. The variation in number of waypoints is indicated by the distribution shown in figure 5. In our analysis, a large majority (over 75%) of birds used 10 waypoints or fewer.

It is likely that this misestimates the total number of memorized waypoints, since locations that are visited only irregularly are not identified, while conversely some waypoints are identified in such close proximity to each other that they are highly unlikely to be independent but instead represent spatially extended waypoints. In addition, temporal misalignments between different flights as a result of factors external to the navigation exercise (such as the presence of other pigeons or predators) can result in true waypoints not being identified. However, this result demonstrates that, in most birds, almost all the repeated structure of the flight trajectories is contained in a small number of waypoints. Sample sizes were too small to detect any significant differences in the waypoint use at different release sites, but we note that the median number of waypoints at each site were: Bladon Heath, 9; Church Hanborough, 10.5; Horspath, 6; Weston Wood, 5.

## 4. Discussion

We have presented a GP model for the distribution of flight trajectories flown by a pigeon from a familiar release site. This was based on the observed tendency of pigeons to form habitual routes over a series of releases from the same site, which manifested itself in the increasing predictive power of our model with increasing release number.

The model we have presented provides an easily extensible and adjustable model for making quantitative predictions about future flight paths based on observations of the past. By providing a probability distribution over flight paths, it creates a framework to compare hypotheses by formalizing the comparison as a model selection problem. In this paper, we have shown how this approach can be used to identify navigational waypoints, selecting both the number of waypoints and their locations by maximizing the predictive power of a subset of the observed data. This provides an algorithmic and objective mechanism for identifying salient locations based only on observed flight paths, without consideration of the landscape. The landscape can thus be independently classified into regions most likely to contain important visual cues.

Our method shows that the repeated structure in flight trajectories of experienced birds is contained in a relatively small number of informative regions. Under the hypothesis that these birds navigate home using visual landmarks, it is highly probable that these maximally informative regions correspond to the use of those memorized visual landmarks, though it is important to note that the location of the waypoint and the location of the corresponding landmark need not be identical. Waypoints may be associated with visual cues at a range of distances. For example, a bird may learn to change its heading when a large distant feature becomes visible on the horizon, creating a waypoint within the flight paths with no proximate landmark. It should be stressed that our algorithm detects waypoints within the flight paths as a proxy for the use of visual landmarks rather than identifying the specific visual feature directly. Therefore, in cases where the landmark is not in the close vicinity of the waypoint, it will be difficult or impossible to determine the specific visual cue associated with that waypoint. Nonetheless, the close proximity of many waypoints to striking visual features revealed by visual inspection of figures 2 and 4 suggests that many waypoints are associated with landmarks in close proximity.

The identified waypoints thus provide a snapshot of the types of landscape that inform or constrain the pigeons' navigation from a familiar release site to the home loft. In some cases, the specific feature of the landscape is unclear from inspection alone, and we must wait for a more in-depth analysis of the landscape before we can judge what characteristic the bird is using to identify that landmark. In many cases, however, there are clear, visually conspicuous features, especially sharp discontinuities in the landscape. Examples of these include the boundaries between terrain types, such as the edges of forests and villages, or roads which represent a sharp break in an otherwise rural landscape. As discussed, with reference to the previous work of Wallraff [19], some of these features may be selected for initially non-navigational reasons associated with the bias of initial flights from unfamiliar areas or through non-navigational pressures such as risk of predation.

A particularly striking finding is that most pigeons form few or no waypoints within urban areas even if they are forced to cross them. This is surprising on some levels since urban areas are rich in visual structure. Nonetheless, this supports the work by Wiltschko *et al.* [20], who found that pigeons showed no evidence of habitual route formation over the information-rich environment of urban Frankfurt (Germany). It is also in line with the findings of Lau *et al.* [21], who observed that high visual information densities were associated with behavioural switches towards more disordered flight patterns. A number of alternative explanations are consistent with this pattern of behaviour. These can be broadly categorized into two types. Either the urban landscape negatively affects the pigeons' ability to effectively memorize and relocate waypoint locations, or the high density of available information negates the need to memorize very precise locations. Within the first category sit the following explanations. Pigeons may have a characteristic visual scale. The visual pattern of urban areas may be informative only below this scale, thus they may appear largely uniform to the pigeon. Alternatively, the pigeons may experience something like a sensory ‘overload’. The sheer quantity of visual information in the urban areas may overwhelm the pigeons' ability to memorize patterns, which would have evolved to recognize patterns in more sparsely featured environments. The encounter rate with non-navigational factors is potentially greater in urban areas, which might cause the pigeon to be distracted from relocating its memorized waypoints. Within the second category lies the possibility that, in the urban area, the pigeon is able to access a greater amount of visual information at a distance, since the average height and density of construction within these regions are higher than in rural areas. With access to this information, the pigeon may be able to successfully determine its position and homeward course without revisiting a very specific waypoint location, thus removing the possibility of discovering the use of waypoints along the route of the flight path. The same effect would be observed if the pigeon had a large and redundant set of waypoints from which to choose on each flight, removing the necessity to return to a specific location each time.

The technique we have demonstrated is applicable to any situation where a level of route habituation has been developed in a repeated navigation exercise, which could potentially be through known geo-stationary waypoints. It could therefore be more widely applied as a general method for locating important regions in animal movement paths wherever habitual movement patterns occur, such as repeated feeding grounds within migratory routes or in learning more about the encoding of repeated patterns of human movement.

### 4.1. Experimental methods

The data used in this paper were collated from two previous studies [7,14]. All experiments followed previously established protocols [22]. Every bird was released a total of 20 times from its selected release site. The flight trajectory was recorded using a micro-GPS logging device attached to the bird's back, and downloaded once the bird was recovered at the home loft. The device recorded the bird's position at a rate of 1 Hz and was accurate to within 5 m.

The locations of the four release sites are indicated in figure 4; see [7] for further details of the experiments at Bladon Heath and Church Hanborough, and [14] for details of experiments at Horspath and Weston Wood.
